# Reactive sulfur species (RSS): possible new players in the oxidative metabolism of plant peroxisomes

**DOI:** 10.3389/fpls.2015.00116

**Published:** 2015-02-25

**Authors:** Francisco J. Corpas, Juan B. Barroso

**Affiliations:** ^1^Group of Antioxidants, Free Radicals and Nitric Oxide in Biotechnology, Food and Agriculture, Department of Biochemistry, Cell and Molecular Biology of Plants, Estación Experimental del Zaidín, Consejo Superior de Investigaciones CientíficasGranada, Spain; ^2^Group of Biochemistry and Cell Signaling in Nitric Oxide, Department of Biochemistry and Molecular Biology, University of JaénJaén, Spain

**Keywords:** nitric oxide, nitrosative stress, oxidative stress, peroxisomes, reactive nitrogen species

Peroxisomes are ubiquitous organelles with a notable oxidative metabolism. In plants, these subcellular compartments have been shown to be involved in the metabolism of reactive oxygen and nitrogen species (ROS and RNS), whose components, hydrogen peroxide and nitric oxide (NO), are important molecules involved in signaling processes. The presence of new elements in plant peroxisomes such as glutathione reductase (GR), sulfite oxidase (SO), glutathione (GSH), and *S*-nitrosoglutathione (GSNO) indicates the involvement of these organelles in the sulfur metabolism. This could suggest the participation of a new family of molecules designated as reactive sulfur species (RSS) which will possibly provide new functions for peroxisomes.

## Critical view

Peroxisomes are remarkable subcellular compartments given their simple morphology (granular/fibrillar matrix bounded by a single membrane) which does not reflect the complexity of their enzymatic composition (Hayashi et al., 2000; Baker and Graham, 2002; el Río et al., 2006). In plant cells, peroxisomes are involved in the photorespiration cycle, fatty acid β-oxidation, the glyoxylate cycle, and the metabolism of ureides (Corpas et al., 1997; Baker and Graham, 2002; el Río et al., 2006; Hu et al., 2012), thus indicating that these organelles play a role in key physiological processes such as seed germination, plant development, fruit ripening, and senescence. Plant peroxisomes have been shown to be a source of ROS including molecules such as superoxide radicals (O^·−^_2_), hydrogen peroxide (H_2_O_2_), and RNS (el Río et al., 2006; el Río, 2011; Corpas et al., 2013; Corpas and Barroso, 2014b). RNS include NO and related molecules such as peroxynitrite (ONOO^−^) and GSNO which are characterized by a broad spectrum of physiological/pathological activities. Both these molecular families (ROS and RNS) include radical molecules containing an unpaired electron as well as non-radical molecules and can also have dual effects depending on their cellular concentration. Thus, H_2_O_2_ and NO at low concentrations can function as signal molecules in the cell or may cause damage to cell components when overproduced as a consequence of adverse conditions (Valderrama et al., 2007; Chaki and Luque, 2011; Signorelli et al., 2013).

Sulfur (S) is an essential mineral for plant growth and development (Leustek and Saito, 1999; Rausch and Wachter, 2005; Hawkesford and De Kok, 2006). It is present in thiamin (B1) and pantothenic acid (B5) vitamins, amino acids (cysteine and methionine), biotin and molybdenum cofactors, and prosthetic groups (Leustek and Saito, 1999) and also in secondary sulfur compounds (polysulfides, glucosinolates, and phytochelatins). In addition, other organic sulfur compounds, such as thiols, GSH, GSNO, and sulfolipids, play an important role in physiological processes and plant stress conditions (Brychkova et al., 2007; Münchberg et al., 2007). In animal cells, the gas hydrogen sulfide (H_2_S) has been shown to be generated from L-cysteine by the pyridoxal-5′-phosphate-dependent enzyme. Thus, endogenous H_2_S can act as a neuromodulator in rat brain (Abe and Kimura, 1996). In higher plants, recent evidence indicates that H_2_S is actively involved in the regulation of ethylene-induced stomatal closure and also interacts with H_2_O_2_ to regulate the plasma membrane Na^+^/H^+^ antiporter system under salinity stress (Hou et al., 2013; Li et al., 2014). The term RSS has been proposed in order to designate a group of sulfur-related molecules that are formed *in vivo* under oxidative stress conditions in animal systems (Giles et al., 2001, 2002; Jacob et al., 2004). These molecules include thiyl radicals (RS·), disulfide-S-oxides [RS(O)_2_SR] and sulfenic acids (RSOH). Thus, high cellular GSH concentrations in an oxidative environment and the decomposition of *S*-nitrosothiols generate disulfide-S-oxides (Tao and English, 2004). These mechanisms can modulate the function of sulfur proteins throughout the redox status of biological thiols (Jacob and Anwar, 2008). Accordingly, disulfide formation is an important cysteine redox reaction in many proteins that affects its function, with thioredoxins and peroxiredoxins being good examples.

**Figure 1:**
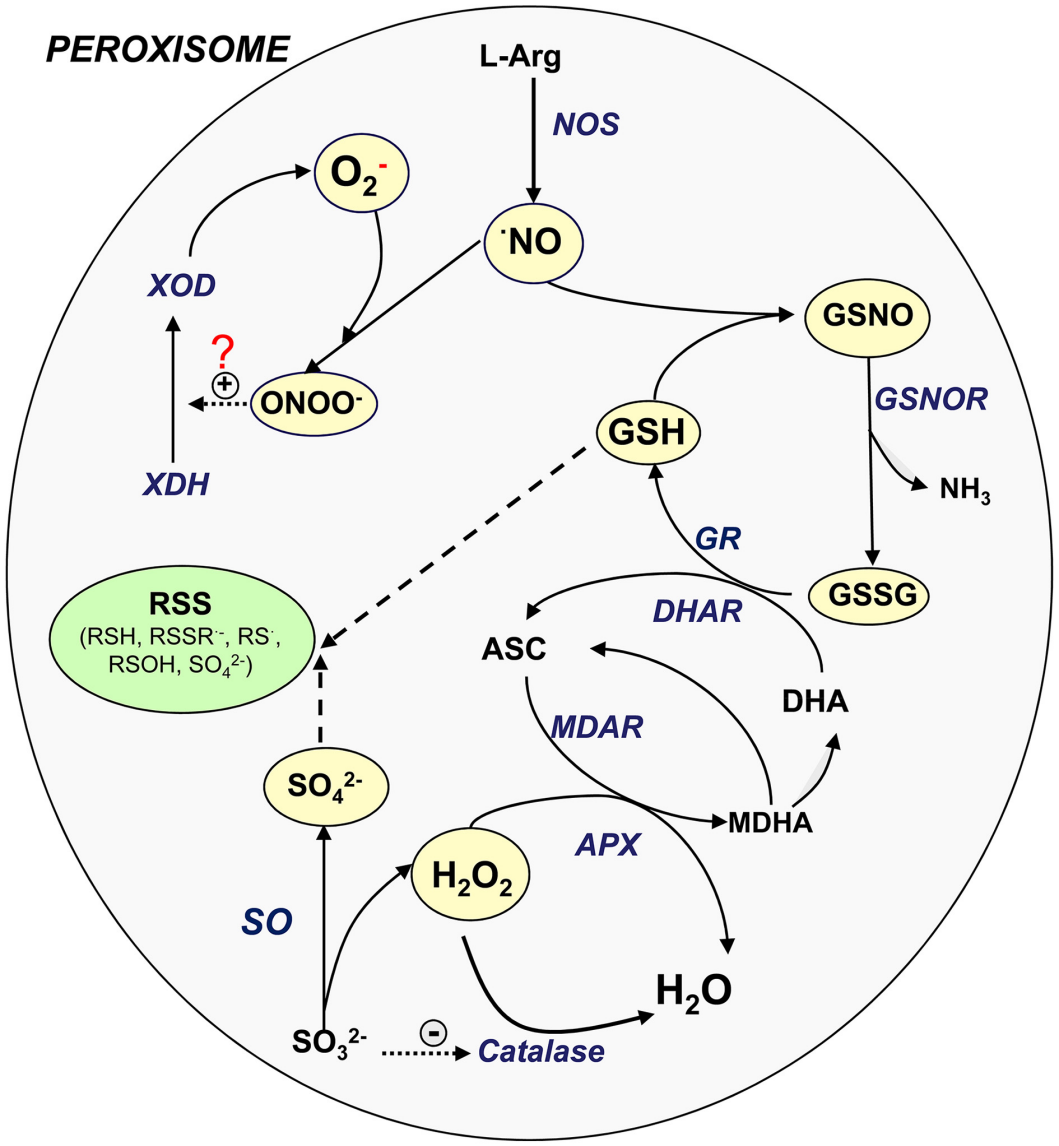
**Signaling cross-talk between NO, ROS, and RSS**. Model of the interaction amongst the different ROS, RNS, and sulfur metabolism into plant peroxisomes. ASC, ascorbate, reduced form; DHA, ascorbate, oxidized form (dehydroascorbate); GSH, glutathione, reduced form; GSNOR, nitrosoglutathione reductase; GSSG, glutathione, oxidized form; NO, nitric oxide; NOS, L-arginine-dependent nitric oxide synthase; MDAR, monodehydroascorbate reductase; ONOO^−^, peroxinitrite; SO, sulfite oxidase; XDH, xanthine dehydrogenase; XOD, xanthine oxidase; RSS, reactive sulfur species; RSH, thiol; RSSR^·−^, disulfide radical; RS·, thyl radical; ROSH, sulfenic acid; SO^2−^_4_, sulfate.

In plant peroxisomes, the presence of important sulfur compounds such as GSH (non-enzymatic antioxidants) (Jiménez et al., 1997; Müller et al., 2004) and GSNO (transport and storage vehicle for NO) has been demonstrated (Barroso et al., 2013). Furthermore, the presence of enzymes such as GR (Jiménez et al., 1997; Romero-Puertas et al., 2006), *S*-nitrosoglutathione reductase (GSNOR) (Reumann et al., 2007; Barroso et al., 2013) and SO (Eilers et al., 2001; Nakamura et al., 2002; Nowak et al., 2004; Hänsch and Mendel, 2005) involved in the sulfur metabolism has also been reported. These new insights lead us to suggest that peroxisomes may play a role in the RSS metabolism, as has been demonstrated for ROS and RNS. Figure 1 shows the potential interactions among the different ROS, RNS, and sulfur-containing compounds in peroxisomes. NO is generated by L-arginine-dependent nitric oxide synthase (NOS) activity (Corpas and Barroso, 2014a) which can react with superoxide radicals generated by xanthine oxidase to form peroxynitrite (ONOO^−^). This RNS is a highly oxidant compound capable of catalyzing the conversion of xanthine dehydrogenase to xanthine oxidase (Corpas et al., 2008) or inducing protein nitration (Radi, 2013). NO can also react with GSH to form GSNO which can be decomposed by GSNOR activity through the generation of GSSG (oxidized form) and NH_3_. GSSG is reduced by GR as a component of the ascorbate-glutathione cycle. H_2_O_2_, which is mainly generated by flavin-oxidases, is decomposed either by catalase or ascorbate peroxidase (APX). SO catalyzes the conversion of sulfite to sulfate with the concomitant generation of H_2_O_2_ (Hänsch et al., 2006). It has been reported that low concentrations of sulfite inhibit catalase activity (Veljović-Jovanović et al., 1998), which could therefore be a means of regulating both enzymes.

In this context, the interactions of ROS, RNS and possibly RSS components in plant peroxisomes open up new challenges and a new area of research to determine the biochemical interactions and potential functions of these reactive species of oxygen, nitrogen and sulfur in peroxisomes, some of which play a very important role as signaling molecules in physiological and phyto-pathological processes (Yamasaki, 2005).

### Conflict of interest statement

The authors declare that the research was conducted in the absence of any commercial or financial relationships that could be construed as a potential conflict of interest.
